# Macrophage Contact Dependent and Independent TLR4 Mechanisms Induce β-Cell Dysfunction and Apoptosis in a Mouse Model of Type 2 Diabetes

**DOI:** 10.1371/journal.pone.0090685

**Published:** 2014-03-03

**Authors:** Helena Cucak, Christopher Mayer, Morten Tonnesen, Lise Høj Thomsen, Lars Groth Grunnet, Alexander Rosendahl

**Affiliations:** 1 Hagedorn Research Institute, Department of Diabetic Complication Biology, Måløv, Denmark; 2 Department of Incretin & Islet Biology, Måløv, Denmark; Heart Research Institute, Australia

## Abstract

Type 2 diabetes (T2D) is evolving into a global disease and patients have a systemic low-grade inflammation, yet the role of this inflammation is still not established. One plausible mechanism is enhanced expression and activity of the innate immune system. Therefore, we evaluated the expression and the function of the toll-like receptor 4 (TLR4) on pancreatic β-cells in primary mouse islets and on the murine β-cell line MIN6 in the presence or absence of macrophages. Diabetic islets have 40% fewer TLR4 positive β-cells, but twice the number of TLR4 positive macrophages as compared to healthy islets. Healthy and diabetic islets respond to a TLR4 challenge with enhanced production of cytokines (5–10-fold), while the TLR4 negative β-cell line MIN6 fails to produce cytokines. TLR4 stimulation induces β-cell dysfunction in mouse islets, measured as reduced glucose stimulated insulin secretion. Diabetic macrophages from 4-months old mice have acquired a transient enhanced capacity to produce cytokines when stimulated with LPS. Interestingly, this is lost in 6-months old diabetic mice. TLR4 activation alone does not induce apoptosis in islets or MIN-6 cells. In contrast, macrophages mediate TLR4-dependent cell-contact dependent (3-fold) as well as cell-contact independent (2-fold) apoptosis of both islets and MIN-6 cells. Importantly, diabetic macrophages have a significantly enhanced capacity to induce β-cell apoptosis compared to healthy macrophages. Taken together, the TLR4 responsiveness is elevated in the diabetic islets and mainly mediated by newly recruited macrophages. The TLR4 positive macrophages, in both a cell-contact dependent and independent manner, induce apoptosis of β-cells in a TLR4 dependent fashion and TLR4 activation directly induces β-cell dysfunction. Thus, targeting either the TLR4 pathway or the macrophages provides a novel attractive treatment regime for T2D.

## Introduction

The global epidemic of Type 2 diabetes (T2D), tied to the rising obesity rate is considered to be one of the major public health problems of the 21st century and the fifth leading cause of death worldwide [1], [2].

T2D is characterized by defective insulin secretion from the pancreatic β-cells and diminished insulin sensitivity in peripheral tissues leading to hyperglycemia [2]. The metabolic overload in these patients triggers a low grade chronic inflammation resulting in a progressive loss of pancreatic β-cells [3]. There is an intimate relationship between the immune and metabolic response systems with overlapping metabolic and inflammatory signaling and sensing pathways. Factors such as lipids and cytokines provide cross-talk between inflammatory and metabolic signaling pathways that contribute to the risk of developing diabetes. For example, overproduction of IL-1β in adipose tissue is an important feature of obesity and contributes significantly to insulin resistance [4]. In human and animal models, elevated free fatty acid (FFA) levels have been observed in obesity and T2D [4], [5] where toll-like receptors (TLRs) are thought to participate in sensing extracellular FFA levels. TLRs are a family of pattern-recognition receptors that play a critical role in the innate immune system by activating pro-inflammatory signaling pathways in response to microbial products [6]. TLR4 is a subclass of TLRs that can be activated by lipopolysaccharide (LPS) and by nonbacterial agonists, such as saturated fatty acids [7]. TLR4 signals via two distinct pathways, a MyD88 dependent or a MyD88 independent pathway. The Myd88 dependent pathway activates the transcription factor NFκB that in turn leads to the transcription of several pro-inflammatory genes, resulting in the production of different pro-inflammatory cytokines such as IL-6, IL-1β and TNFα. The activation of TLR4 by FFAs and subsequent upregulation of intracellular inflammatory pathways establish a link between the innate immunity and diet induced obesity and insulin resistance. Indeed numerous studies consistently demonstrate that TLR4 deficiency protects against the development of diet-induced obesity and insulin resistance [7]–[9].

Macrophages are a heterogeneous population of cells expressing various general markers, such as F4/80, CD68, CD11b, and Ly6c, in a heterogeneous fashion, dependent on tissue of origin, maturation, and activation degree [18]. The local tissue environment provides macrophages with various stimuli creating an array of distinctly activated cells. Particularly, in the islets of Langerhans two distinct macrophage subsets based on the expression of CD68 and F4/80 are present. The CD68^+^F4/80^−^ macrophage population invades the diabetic islets and expresses an M1-like phenotype characterized by enhanced levels of CD11c over the M2-like marker CD206 [18]. The majority of the splenic cytokines in response to the elevated glucose in early and late diabetes are macrophage-derived where the expanded CD68^+^F4/80^−^ subpopulation is the predominant producer. The diabetic macrophages acquire an enhanced capacity to synthesis pro-inflammatory cytokines as well as tissue remodeling cytokines, such as TGF-β [18].

Even if TLRs are mainly expressed on antigen presenting cell such as macrophages and dendritic cells, TLR4 has been identified on several other cell types including pancreatic β-cells [10], as well as other insulin sensitive tissues such as adipocytes and muscle cells [7], [11]. Whereas the role of TLR4 signaling in insulin resistance is a well-established concept the specific roles of TLR4 expressing cells in respect to the pathogenesis of T2D are still an unexplored area.

Until recently TLR4 expression in islets has only been examined under normal conditions and not with respect to diabetes development. However, a study by Ladefoged et al showed increased expression of TLR4 mRNA accompanied by pro-inflammatory cytokine release in islets with the development of T2D [12]. Whether this pro-inflammatory milieu might originate either from the β-cells themselves, infiltrating macrophages [13] or other cells in the islets is still an open question. To assess the basis of TLR4 signaling in the pathogenesis of T2D, we compared the TLR4 mediated pro-inflammatory response of islets and spleen in diabetic db/db and db/+ mice. This mouse strain has a defective leptin receptor and develops significant obesity spontaneously. Furthermore, the mice also progress into classical signs of T2D such as β-cell dysfunction, elevated blood glucose and HbA1c as well as severe insulin resistance [14]. This is in contrast to the many commonly used diet induced obesity models which all lead to obesity and moderately enhanced blood glucose, but never develop the classical characteristics of T2D [15], [16]. Further, since inflammation triggered β-cell apoptosis has been suggested as a central component of the development and progression of T2D [17], we explored the ability of diabetic and non-diabetic TLR4 activated splenocytes to induce β-cell apoptosis. Our data suggests that the TLR4 signaling pathways are indeed present and active in the islets of Langerhans. The pathophysiological consequence of the enhanced TLR4 activity in T2D appears to be enhanced induction of β-cell apoptosis mediated by both direct cell contact dependent and independent mechanism.

## Materials and Methods

### Ethics Statement

All animal experiments were approved by the Copenhagen Animal Ethics Committee and performed according to their recommendations.

### Mice

Male homozygous diabetic (db/db) and non-diabetic control littermates (db/+) on C57BL/Ks background were purchased from Taconic at 6 weeks of age and housed according to standard rules at the Novo Nordisk A/S animal facility (Måløv, Denmark). Mice were given free access to NIH-31 M rodent diet (Harlan Laboratories, Boxmeer, Netherlands) and tap water and maintained at 22–24°C.

### Cell Culture & Reagents

Mouse MIN6 pancreatic β-cells were cultured in DMEM (25 mM glucose, 2 mM l-glutamine, and 1 mM sodium pyruvate) supplemented with 10% FBS and 100 µM β-mercaptoethanol at 37°C and 5% CO_2_. LPS used was purchased from Sigma (L5543, Lot: 110M4090). Due to differences in LPS activity from batch/LOT numbers, full dose-response evaluations in each cell type was performed and a cell specific concentration equivalent to an estimated EC_80_ of LPS was used in all functional experiments. Free fatty acid (FFA) is palmitate (Sigma P5585) in a 50% solution with Oleic acid (Sigma O1383). 50 mM solution of palmitate in 90% ethanol was heated to 60°C. Oleate was dissolved in 90% ethanol and heated to 60°C. They were mixed in a 1∶1 solution, vortexed and dissolved into the right concentrations just before use.

### Isolation of Pancreatic Islets and Spleens and Generation of Single Cell Suspensions

8 week, 16 and 24 week old db/db and db/+ mice were killed by cervical dislocation and pancreas and spleen were removed. For islet isolation, pancreas from 8 weeks old mice was transferred to cold collagenase (grade II 300 units/ml, MP Biomedical), shaken in the Grant/Edmund S25 thermoshaker at 200 strokes/min at 37°C for 10 min, transferred to a fresh vial with 75 units/ml collagenase, shaken for an additional 5 min and the supernatants were collected. The procedure was repeated several times. Finally, the supernatants were washed three times in HBSS supplemented with 10 mmol/l HEPES, 5 mmol/l NaHCO3, 1% P/S and 0.5% fetal calf serum (FCS) and allowed to sediment for 5 min between each wash, and finally left on ice before hand-picking.

Female NMRI mice (Taconic) at 10–13 weeks of age were killed by cervical dislocation. Liberase mix (117 units/mL Liberase TL (Roche); 25 mM CaCl2 (Sigma-Aldrich); 14 units/µL DNaseI (BioNordika) diluted in HBSS Ca2+/Mg2+ (Gibco) was injected into the pancreas via the common bile duct. The pancreas was dissected followed by digestion at 37°C for 18 min. Collagenase activity was stopped with stop buffer (3 g/l BSA (Sigma-Aldrich); 0.5 g/l D-glucose (Sigma) diluted in HBSS Ca2+/Mg2+ (Gibco). The digest was filtered through a 400 µM cell strainer and after three washes; islets were hand-picked from purified islet fractions made with 100 and 70 µM cell strainers.

Isolated islets were cultured in complete RPMI 1640 (RPMI + Glutamax (Gibco) added 10% (vol/vol) FBS (Invitrogen), 1% (vol/vol) penicillin/streptomycin (Gibco), 20 mM Hepes (Gibco)) in a 5% CO_2_ humidified atmosphere at 37°C.

A single cell suspension from the islets were generated by re-suspension in trypsin-EDTA in HBSS w/o Ca^2+^/Mg^2+^ at 37°C and pipetting up and down for 3 minutes followed by wash with FACS buffer (PBS, 10% FCS, 2 mM EDTA).

Single splenocytes suspensions were generated by pressing spleens from db/db and db/+ mice through a 70 µm cell strainer after which the erythrocytes were lysed with cell lysis buffer.

### Cytokine Production from Islets

The isolated islets were rested overnight in complete RPMI media and cultured in 96-well plates at concentration of 10 islets/well in the absence or presence of LPS (0.1 µg/ml, 1 µg/ml and 10 µg/ml). After 24 h, culture supernatants were collected and analysed for IL-6, KC, MCP-1, RANTES and VEGF on the Bio-Plex 200 (Biorad) using the Milliplex map kit (Mouse cytokine/chemokine magnetic bead panel) (Millipore) according to the manufacturer’s description.

### Glucose-stimulated Insulin Secretion in NMRI Islets

Thirty islets per well were transferred to an extracellular-matrix-coated 96-well plate (Biological Industries) in complete RPMI1640 and incubated overnight in a 5% CO_2_ humidified atmosphere at 37°C. Medium was removed and NMRI islets were washed in KRBH (115 mM NaCl; 4.7 mM KCl; 2.6 mM CaCl_2_; 1.2 mM KH_2_PO_4_; 1.2 mM MgSO_4_; 5 mM NaHCO_3_ (all from Merck); 1% penicillin/streptomycin (vol/vol) (Gibco); 20 mM Hepes (Gibco); 0.02 mM Glutamin (Gibco); 0.2% (wt/vol) BSA (Sigma), pH 7.4). Islets were then incubated in KRBH added 3 mM D-glucose for 0.5+1 h and KRBH added 15 mM D-glucose with/without LPS or 150 pg/ml IL-1β and 5 ng/ml IFNγ for 1 h. Buffer was collected from the last 3 mM and 15 mM incubations, filtered through a 96-well filter plate (Multiscreen-DV, Millipore) and stored at −20°C until analysis. Each experiment was normalized to the insulin release at 15 mM ( = 100%). Thus, when calculating the average from the 3 independent experiments, the variation of the 15 mM stimulation will be 0.15 mM glucose corresponds to the lowest concentration of glucose giving maximum insulin release.

### Measurement of Insulin

Detection of insulin was by luminescence oxygen channeling immunoassay (LOCI). Anti-insulin mAb RDI-TRK2IP10-D6C4 was conjugated to LOCI acceptor beads (PerkinElmer) and another anti-insulin mAb RDI-TRK2IP10-D3E7 (binding to a different epitope) was biotinylated. The assay was conducted in 384-well plates by adding 1 µl of calibrator, control and unknown sample in the wells followed by 15 µl of a mixture of acceptor beads and biotinylated antibody. After 1 h of incubation at 21–22°C, 30 µl of streptavidin-coated donor beads were added and the plates were further incubated for 30 min. The plates were read in an Envision plate reader (PerkinElmer) at 21–22°C, applying a 520–645 nm filter after excitation by a 680 nm laser. The total measurement time per well was 210 ms including a 70 ms excitation time. During the assay the three reactants combine with analyte to form a bead-aggregate-immune complex. Illumination of the complex triggers chemiluminescence from the acceptorbeads which is measured in the EnVision plate reader. The amount of light generated is proportional to the concentration of insulin. The concentration of samples was calculated against a standard curve of rat insulin using a 5 parameter fit. The lower limit of quantification was 0.36 ng/ml.

### Cytokine Detection Assays

Cytokine production from mouse islets or MIN6 cells was performed by treating 10 islets or 30 000 MIN6 cells in 96-well plates in a final volume of 100 µl. For quantification of cytokine production in splenocytes, 500 000 cells were treated in 400 µl in 24-well plates. Cells or islets were treated with LPS for 24 h and supernatants were assayed for IL-6, MCP-1, TNFα and IL-1β by AlphaLISA (Perkin-Elmer, Waltham, MA) according to the manufacturer’s protocols. Experiments were performed in triplicates.

### Measurement of Cell Death

Apoptotic cell death in whole murine islets was determined in duplicates of size-matched islets by the detection of DNA-histone complexes present in the cytoplasmic fraction of cells using Cell Death Detection ELISA^PLUS^ (Roche, Basel, Switzerland). Briefly, 10 islets were lysed in 200 µl lysis buffer for 30 min at room temperature. Lysates were centrifuged for 10 min at 200×g, and 20 µl of the supernatant and 80 µl immuno-reagent (anti-DNA-POD antibody and anti-histone-biotin antibody) were added to streptavidin-coated microtiter plates and incubated for 2 h under shaking conditions (300 rpm) at room temperature. After washing three times with 200 µl incubation buffer, 100 µl ABTS (2,2-azino-bis-3-ethylbenzthiazoline-6-sulfonate) solution was added to each well. Absorbance was measured at 405 and 490 nm.

Apoptotic cell death in MIN6 cells was assayed using the ApoTox-Glo Triplex Assay kit from Promega (Madison, WI). In brief, 30 000 MIN6 cells were seeded in a final volume of 100 µl 96-well plates and then either treated after 24 h or co-cultured or in transwell systems with splenocytes at a ratio of 1∶10 for an additional 24 h before further treatment. The cells were then treated with 10 and 100 ng/ml LPS, 150 pg/ml IL-1β and 5 ng/ml IFNγ or conditioned media from LPS-activated splenocytes (only for single cultured MIN6). After 24 h of incubation, Caspase-Glo 3/7 Reagent was added to the cells and after 30 min of incubation at room temperature, luminescence was measured using the SpectraMax M4 (Molecular Devices). Quantification of caspase 3/7 activity was measured in duplicates.

### Flow Cytometry Analysis

Flow cytometric analysis was performed according to standard procedures. Briefly, cells (pancreatic islet cells or splenocytes) were first blocked for unspecific binding with anti-CD16/CD32 (BD Pharmingen) followed by surface staining of F4/80 and TLR4 (Biolegend). Cells were then fixed and permeabilized using Cytofix/Cytoperm Fixation/Permeabilization Solution Kit (BD Pharmingen) according to manufacturer’s description and then intracellularly stained for CD68 (AbD Serotec) and insulin (R&D Systems).

Intracellular cytokine levels were evaluated in splenocytes cultured for 6 h at 37°C at 10^6^ cells/ml in presence of 10 µg/ml BrefA and 100 ng/ml LPS. Cells were then stained for surface antigens including F4/80 followed by fixation and permeabilization and finally stained for TNFα (eBioscience), IL-6 (Biolegend), IL-1β (e-Bioscience), and CD68. Samples were then acquired on a FACSFortessa equipped with blue, red and violet laser followed by data analysis using FACSdiva software (BD Biosciences).

### Statistical Analysis

Statistical analyses were performed with GraphPad Prism 6 using unpaired two-tailed Student’s t-test or two-way ANOVA with a multiple-comparisons test.

## Results

### TLR4 Expression on Small Non-granular Insulin Producing Cells and CD68^+^F4/80^+^ Macrophages

TLR4 mRNA expression has previously been observed in both human [10] and mouse [12] pancreatic islets with increased expression during disease progression in T2D mouse models. We have previously demonstrated two distinct populations of macrophages infiltrating the diabetic islets based on CD68 and F4/80 expression i.e. CD68^+^F480^+^ and CD68^+^F4/80^−^ subsets [18]. The latter showed an M1-like phenotype and was specifically recruited to diabetic pancreatic islets [18]. To evaluate the expression of TLR4 on these macrophage subsets and non-immune cells, known to under specific circumstances upregulate TLR4 [19], a flow cytometric evaluation of islet derived macrophages and insulin producing cells was performed.

**Figure 1:**
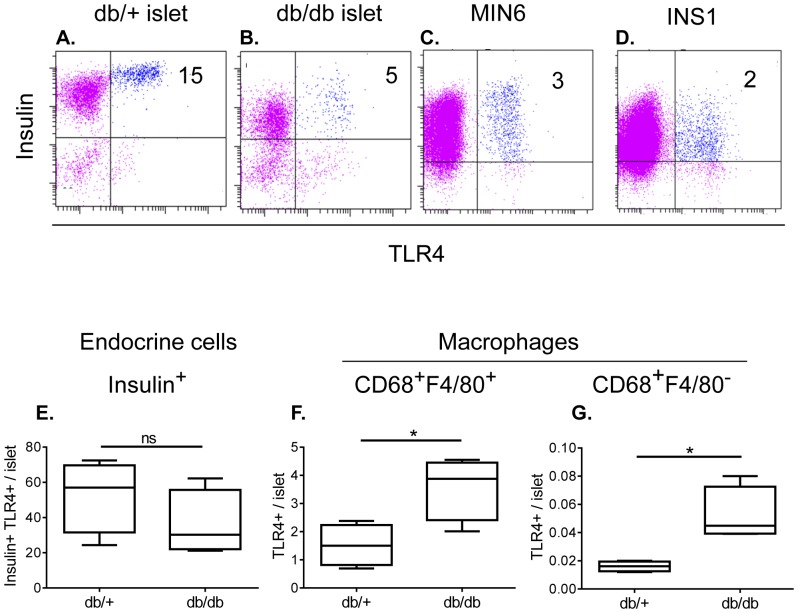
TLR4 expression on cells in islets. Representative flow cytometic analysis showing percentages of insulin^+^TLR4^+^ cells in pancreatic islets of 8 weeks old db/db and db/+ mice, MIN6 and INS1 cells (A-D). The number indicates percentage of cells within the gate. Number of insulin^+^ cells (E), CD68^+^F4/80^−^ (F) and CD68^+^F480^+^ (G) macrophages expressing TLR4 in pancreatic islets of 8 weeks old db/+ and db/db mice. Three independent experiments were performed with a pool of 20 mice in each group per experiment. Unpaired T-test ***p<0.001; **p<0.01; *p<0.05.

Both insulin producing cells and macrophages expressed TLR4 in the islet (Fig. 1A–G). Amongst insulin positive cells, only the small non-granular cells expressed TLR4 (data not shown). Interestingly, a non-significant trend towards reduced frequency of TLR4 positive β-cells was observed in diabetic islets (Fig. 1A, B, E). Both the diabetic and the non-diabetic islets had 10-times more TLR4 positive CD68^+^F4/80^+^ macrophages than CD68^+^F4/80^−^ macrophages (Fig. 1F, G). Importantly, the number of TLR4 positive macrophages increased in diabetic islets within both macrophage sub-populations (Fig. 1F, G). The β-cell lines, MIN6 and INS-1, showed low expression of TLR4 similar to that observed in db/db islets (Fig. 1B–D).

These results demonstrate that the capacity to respond to innate immunity signals mediated through TLR4 is present in the local pancreatic islet milieu. Furthermore, the data suggests that the capacity to respond is modified in diabetes as more TLR4 positive macrophages are present in the diabetic tissue.

### TLR4 Stimulation Directly Interferes with β-cell Function by Inhibition of Insulin Secretion

It has previously been shown that LPS inhibits β-cell insulin gene expression in a TLR4-dependent manner in both isolated islets and β-cell lines [10], [20], [21]. To evaluate if TLR4 agonism directly impairs glucose stimulated insulin secretion (GSIS) mouse islets were exposed to three concentrations of LPS for 24 h (as an agent to mimic TLR4 agonism) in complete media. GSIS was measured after the last 3 mM glucose incubation and the 15 mM glucose challenge as a measurment of β-cell function.

**Figure 2:**
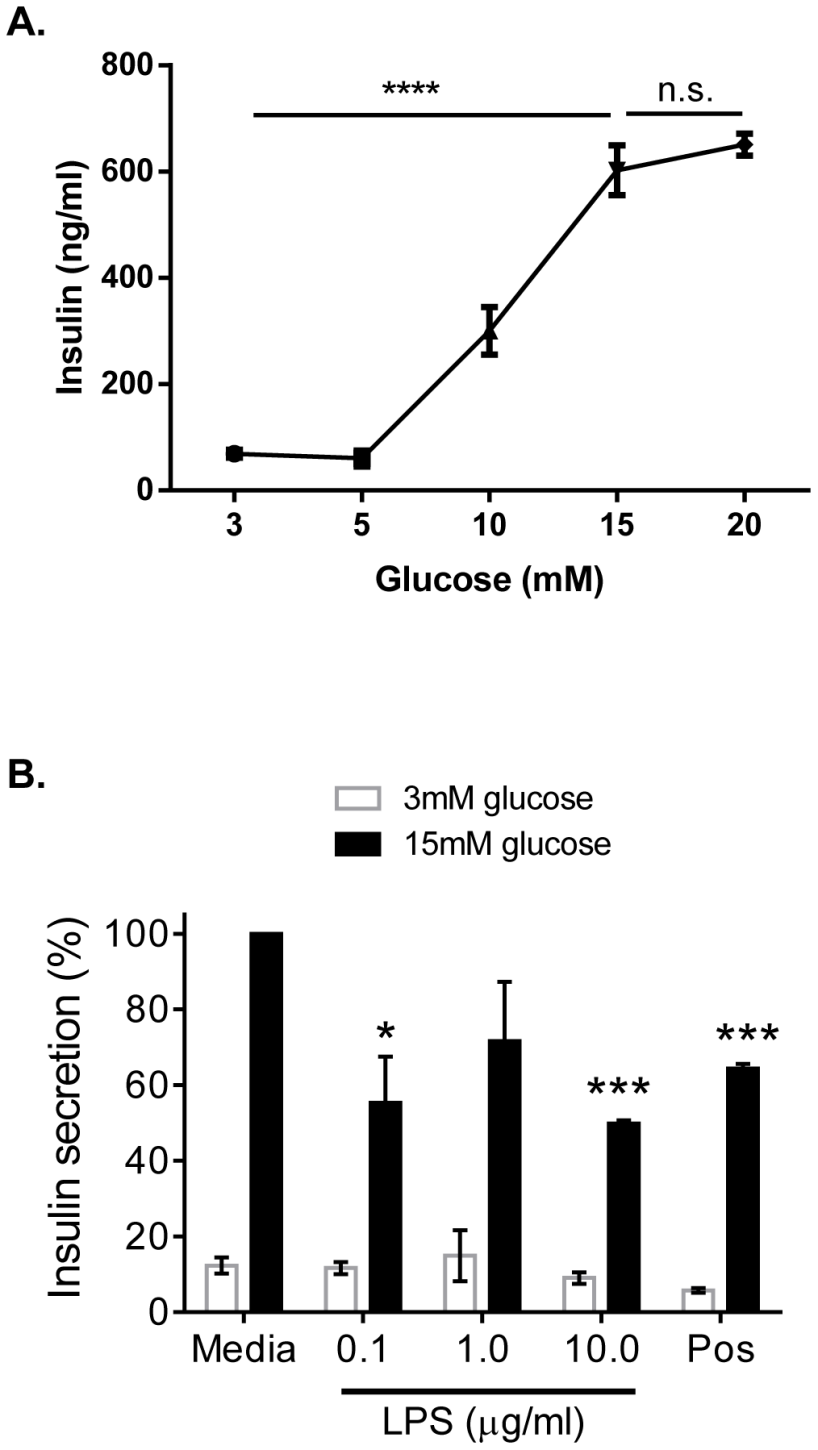
TLR4 reduces glucose stimulated insulin secretion. NMRI islets were exposed to increasing concentrations of glucose and insulin was measured (A). Islets were pre-incubated with LPS for 24 h before the glucose-stimulated insulin secretion was measured using a concentration of 15 mM glucose (B). Data are presented as normalized averages +−/SEM from 3 independent experiments where each experimental was performed in triplicates. Statistical significance was evaluated by an unpaired two-sided parametric t-test assuming equal variance performed on normalized data. *; **; *** represent p<0.05; 0.01; 0.001, respectively, vs. insulin secretion at control islets at 15 mM glucose. **** represent p<0.0001 of insulin secretion at 15 mM versus at 3–10 mM glucose and n.s. represent non-significant using one-way ANOVA.

Stimulation of islets with increasing doses of glucose showed that the lowest dose of glucose providing maximum insulin release was obtained at a dose of glucose equivalent to 15 mM (Fig. 2A). Exposure of islets to LPS for 24 h significantly decreased β-cell function measured as glucose-stimulated insulin secretion (Fig. 2B). A significant effect was obtained already at 0.1 µg/ml of LPS and at 10 ug/ml the effect was similar as to the positive cytokine control included (Fig. 2B).

These data demonstrate that the TLR4 expressed on the islets is functional and that signaling through TLR4 in islets induces β-cell dysfunction measured as a reduction of GSIS.

### Diabetic Islets Show an Increased Pro-inflammatory Response after LPS Stimulation

TLR4 challenge is known to induce a pro-inflammatory cytokine signature from various cells and tissues. As cytokines have been implicated to promote dysfunction of islets in diabetes and the overall significantly enhanced TLR4 expression in diabetic islets compared to healthy islets, the TLR4 induced cytokine signature was determined in isolated islets.

**Figure 3:**
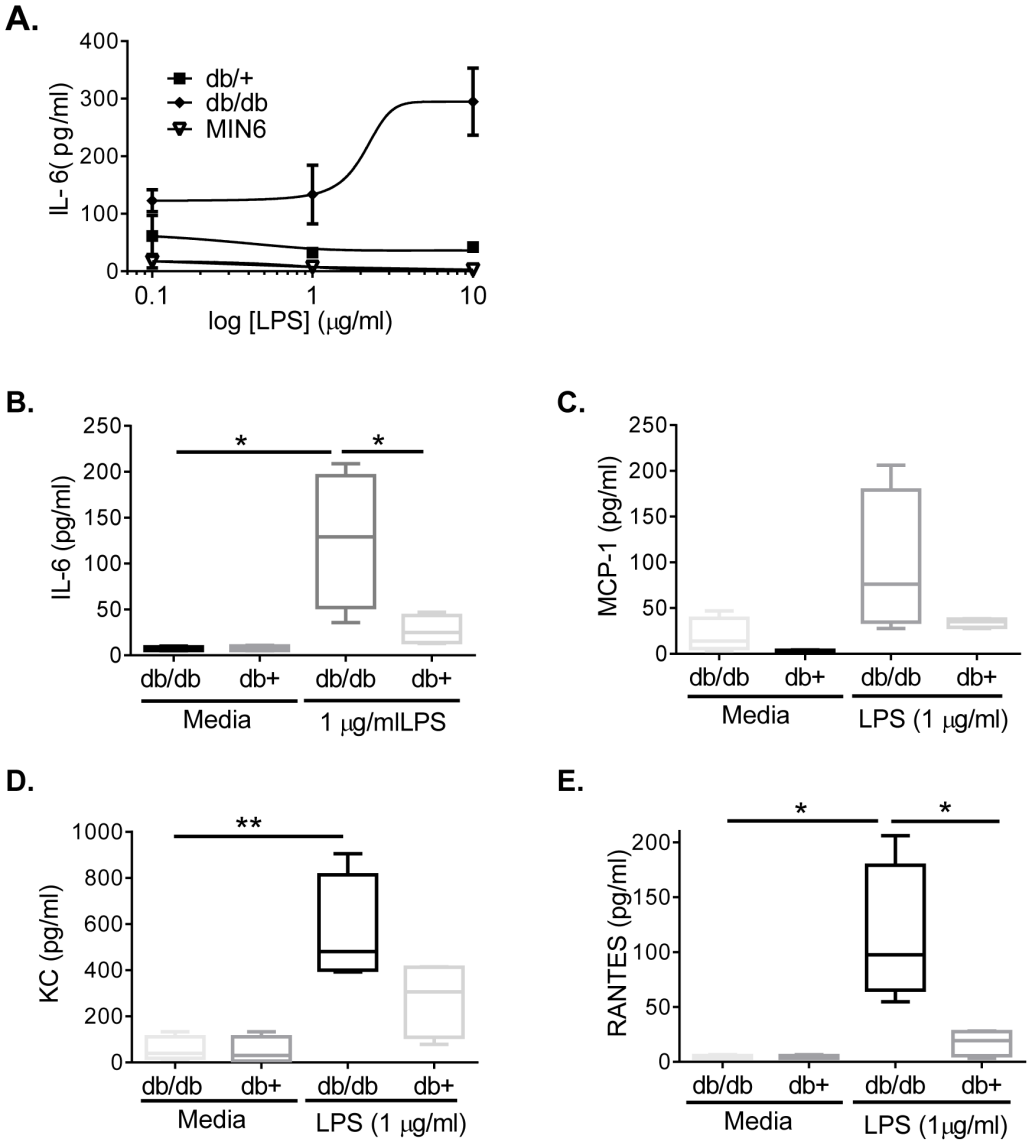
LPS induced cytokine production in islets. Islets from db/+, db/db, and MIN6 cells left untreated or treated with the indicated LPS concentrations for 24 h and media. LPS induced dose-response of IL-6 (A). Release of IL-6 (B), MCP-1 (C), KC (D) and RANTES (E) from db/+ and db/db islets treated with 1 µg/ml LPS or media alone. Data shows mean from 20 mice run in triplicate. All data are shown as mean ± S.E.M. Statistical analysis is derived by student’s T-test*p<0.05; p<0.01; p<0.001.

LPS induced a dose-dependent production of IL-6 in db/db islets with an estimated EC_80_ at 1 µg/ml, but not in db/+ islets or in MIN6 cells (Fig. 3A). In line with the enhanced number of TLR4 positive macrophages in the diabetic islets, a clear and significant pro-inflammatory signature was noted by enhanced levels of IL-6 (Fig. 3B), KC (Fig. 3D), RANTES (Fig. 3E) as well as a non-significant increase of MCP-1 production (Fig. 3C). All cytokines were produced in substantial levels already at a concentration of 100 ng/ml with maximal levels obtained at 1–3 µg/ml LPS (data not shown). FFA stimulation induced a similar pro-inflammatory cytokine signature as LPS, but the magnitude of response was markedly lower than that observed upon LPS challenge (S Fig. 1).

These data suggest that the recruited macrophages in the diabetic islets contribute to create a local inflammatory milieu that shows enhanced responsiveness to activation through the innate TLR4 pathway in T2D.

### Similar Expression of TLR4 in Diabetic and Healthy Systemic Macrophages over Time

It is now well recognized and accepted that T2D is chronic low grade inflammatory disease. For that reason, TLR4 can contribute to the disease progression also in the systemic compartment which then influences the local inflammatory milieu in the pancreatic islet. To address this, expression of TLR4 in the splenic macrophages was determined by flow cytometric analysis.

**Figure 4:**
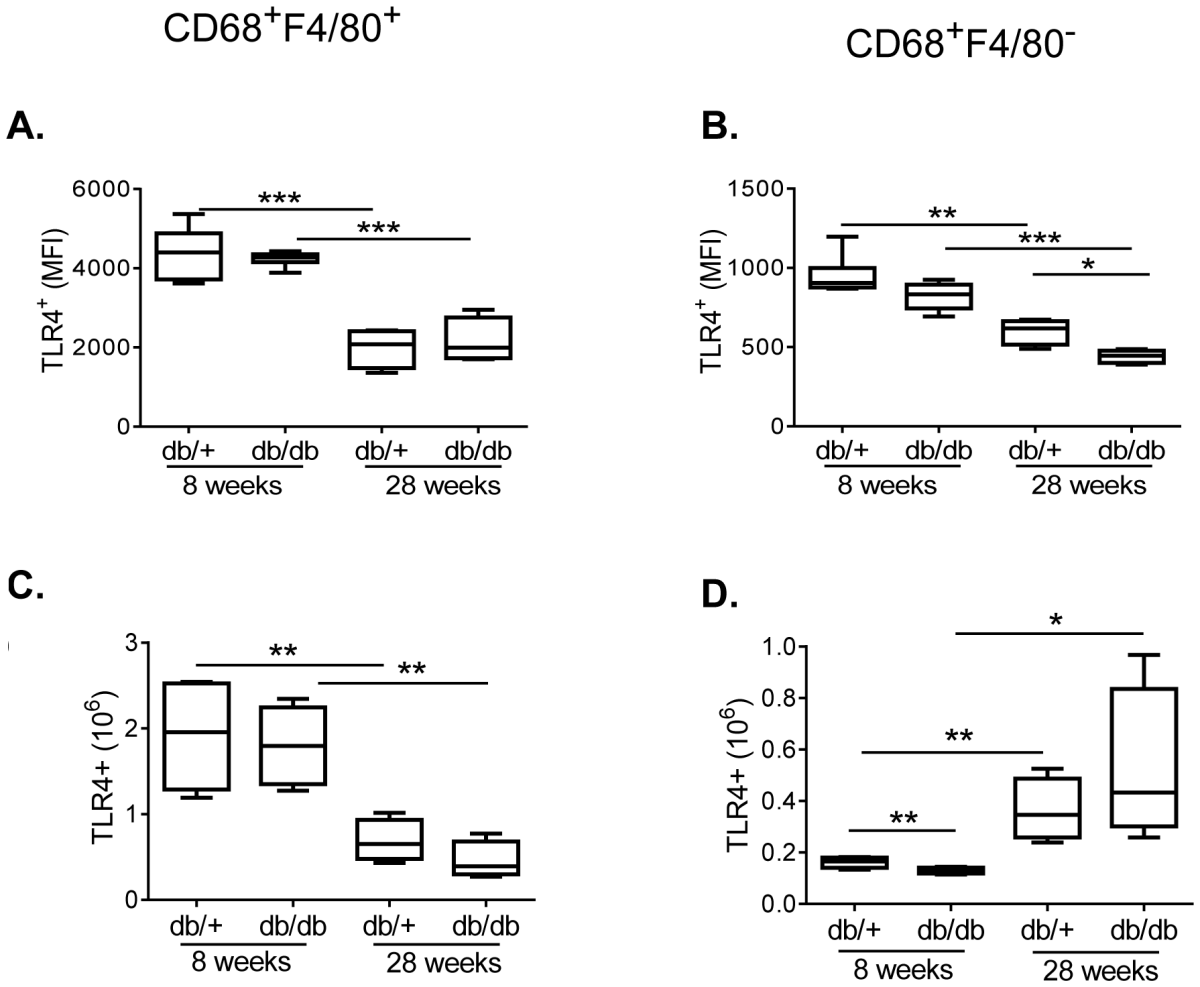
TLR4 expression in spleen. Mean fluorescence intensity (MFI) (A and B) and total number (C and D) of CD68^+^F4/80^+^ and CD68^+^F4/80^−^ macrophages expressing TLR4 at indicated age in db/+ and db/db mice. One representative experiment out of three is shown. Unpaired T-test ***p<0.001; **p<0.01; *p<0.05.

Consistent with the islets, the CD68^+^F4/80^+^ macrophage subset expressed markedly higher levels (4-fold) of TLR4 than the CD68^+^F4/80^−^ macrophages (Fig. 4 A, B). Early in life the number of TLR4 positive CD68^+^F4/80^+^ macrophages was 10-fold higher compared to CD68^+^F4/80^−^ macrophages (Fig. 4 C, D). Importantly, with age the number of TLR4 positive CD68^+^F4/80^+^ macrophages significantly declined, while the number of TLR4 positive CD68^+^F4/80^−^ macrophages increased 2-fold (Fig. 4 C, D). Thus at 7-months of age the expression pattern was similar between the two macrophage subsets (Fig. 4 C, D). Noteworthy, the expression pattern of TLR4 both per cellular level and per subpopulation level was independent of disease.

These data suggests that TLR4 expression is high in early life and lower at later stages, but in contrast to the islets of Langerhans, expression of TLR4 in the secondary lymphoid tissues does not appear to be regulated by diabetes.

### The TLR4 Induced Cytokine Signature is Transiently Aggravated in Diabetic Splenocytes

As the expression of TLR4 showed to be independent of diabetes and showed a negative correlation with age, i.e. progression of the diabetes in the systemic compartment, the capacity to respond to TLR4 was measured by monitoring cytokine levels after LPS challenge at various ages of the mice.

**Figure 5:**
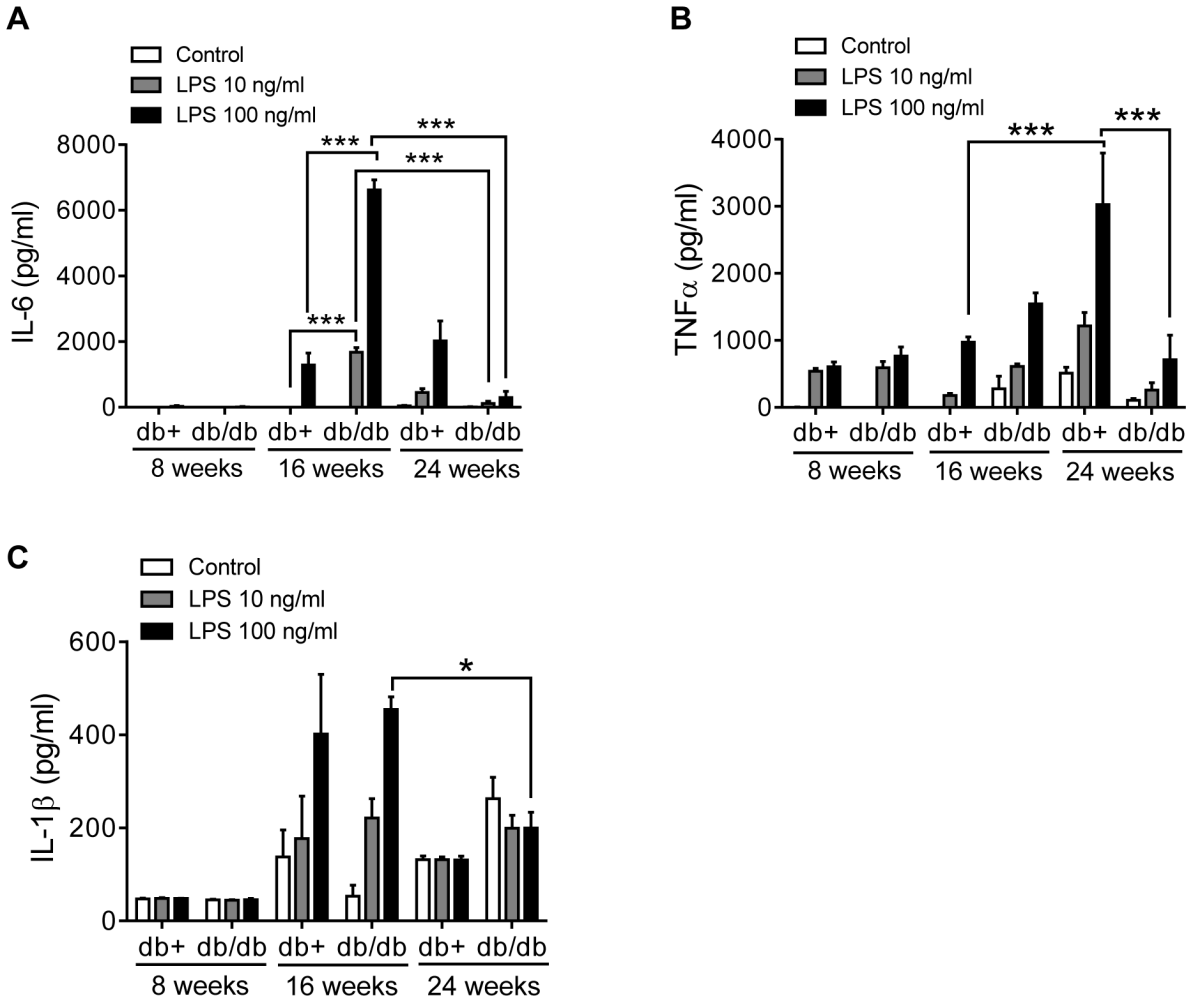
Transiently aggravated TLR4 induced cytokine signature in diabetic splenocytes. Splenocytes from 8, 16 and 24 weeks old mice were treated with LPS and production of IL-6 (A), TNFα (B) and IL-1β (C) was monitored. Data shows one representative experiment with mean ± S.E.M from 4 mice, Statistical evaluation conducted by two-way ANOVA *p<0.05; p<0.01; p<0.001.

IL-6 secretion was minimal in both db/db and db/+ splenocytes at 8 weeks (Fig. 5A). At 16 weeks, IL-6 was significantly increased by both 10 and 100 ng/ml LPS in diabetic splenocytes, whereas healthy splenocytes only responded to 100 ng/ml LPS (Fig. 5A). The level of IL-6 was significantly higher in diabetic compared to the healthy splenocytes at both concentrations (Fig. 5A). Intriguingly, the IL-6 production was significantly lower in the 24 week diabetic compared to the 16-week old diabetic splenocytes, while healthy 24-week old splenocytes produced similar levels as produced at 16 weeks of age (Fig. 5A). TNFα secretion was similar at 8 and 16 weeks in diabetic and healthy splenocytes, whereas a significant increase in TNFα secretion in the LPS-stimulated 24 week healthy but not in the diabetic splenocytes (Fig. 5B) was observed. IL-1β secretion was not increased by LPS at 8 or 24 weeks compared to medium (Fig. 5C). Interestingly, at 16 weeks of age both healthy and diabetic splenocytes responded to LPS by a dose dependent production of IL-1β (Fig. 5C).

These data demonstrate that activation of TLR4 on macrophages induces a pro-inflammatory cytokine signature that depends on the age. Maximum enhanced responsiveness to TLR4 measured as cytokine production occurs at 4-months of age in diabetic mice, while healthy mice appear to have a lower but more prolonged responsiveness reaching at least through 6 months of age.

### Accelerated Acquisition to Produce Cytokines in Diabetic Macrophages

To determine the cellular origin of TLR4-induced cytokines in the spleens, the cellular expression pattern of cytokines after LPS challenge was determined by flow cytometry in diabetic and non-diabetic young and old mice.

**Figure 6:**
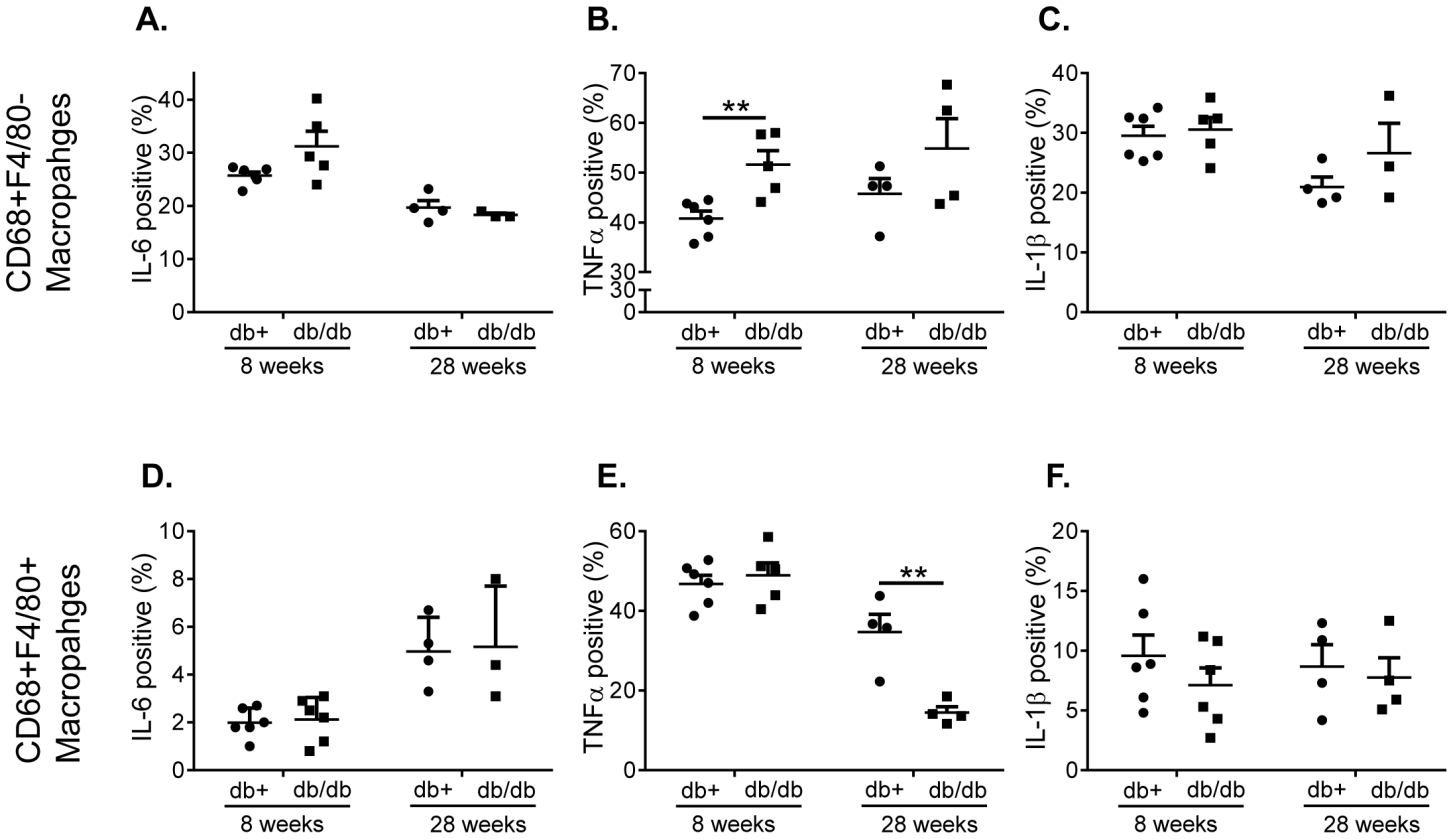
Accelerated acquisition to produce cytokines in diabetic macrophages. Splenocytes were stimulated with 100/ml LPS in the presence of 10 µg/ml Bref-A for 6 h. Intracellular cytokines expression of IL-6 (A, D), TNFα (B, E) and IL-1β (C, F) was then determined in CD68^+^F4/80^−^ and CD68^+^F4/80^+^ macrophages respectively. One representative experiment out of two with at least 4 animals in each is shown. Statistical evaluation using unpaired T-test ***p<0.001; **p<0.01; *p<0.05.

TLR4 challenge did not induce cytokine production in the non-macrophage compartment in the spleen demonstrating that macrophages are the major source of inflammatory cytokines in the spleen in response to TLR4 (data not shown). The CD68^+^F4/80^−^ and the CD68^+^F4/80^+^ macrophages both responded to TLR4 challenge with induction of the pro-inflammatory cytokines IL-6, TNFα and IL-1β (Fig. 6A–F). TLR4 induced TNFα production was similar in both macrophages subsets (Fig. 6B, E). In contrast, the frequency of CD68^+^F4/80^−^ macrophages producing IL-6 and IL-1β was 3–10 fold higher than in CD68^+^F4/80^+^ macrophages (Fig. 6A, C, D, F). In addition, the number of CD68^+^F4/80^−^ macrophages in the spleen is significantly higher than the CD68^+^F4/80^+^ macrophages further demonstrating that the main source of TLR4 induced cytokines are the CD68^+^F4/80^−^ macrophages (data not shown). In early stages of diabetes the frequency and hence number of TNFα producing CD68^+^F4/80^−^ macrophages are higher compared to healthy control mice (Fig. 6B). A similar trend although not reaching significance is evident also after 28-weeks (Fig. 6B). Interestingly, the capacity to produce TNFα appears reduced in CD68^+^F4/80^+^ macrophages at late stage of disease, but this does not translate into reduced number of cells (Fig. 6E, data not shown).

Taken together, these data demonstrate that the cytokines produced in the spleen after TLR4 are mainly coming from the CD68^+^F4/80^−^ macrophages. Further, diabetes appear to accelerate the capacity to produce cytokines in response to TLR4 as maximum levels are reached earlier in life in diabetic splenocytes compared to healthy.

### TLR4 Activated Splenocytes Release Soluble Factors Mediating Apoptosis of β-cells and Islets

As β-cells undergo apoptosis in response to certain cytokines, and a functional TLR4 receptor is shown in the islets that leads to production of cytokines, the capacity for TLR4 to induce apoptosis by soluble factors was evaluated.

**Figure 7:**
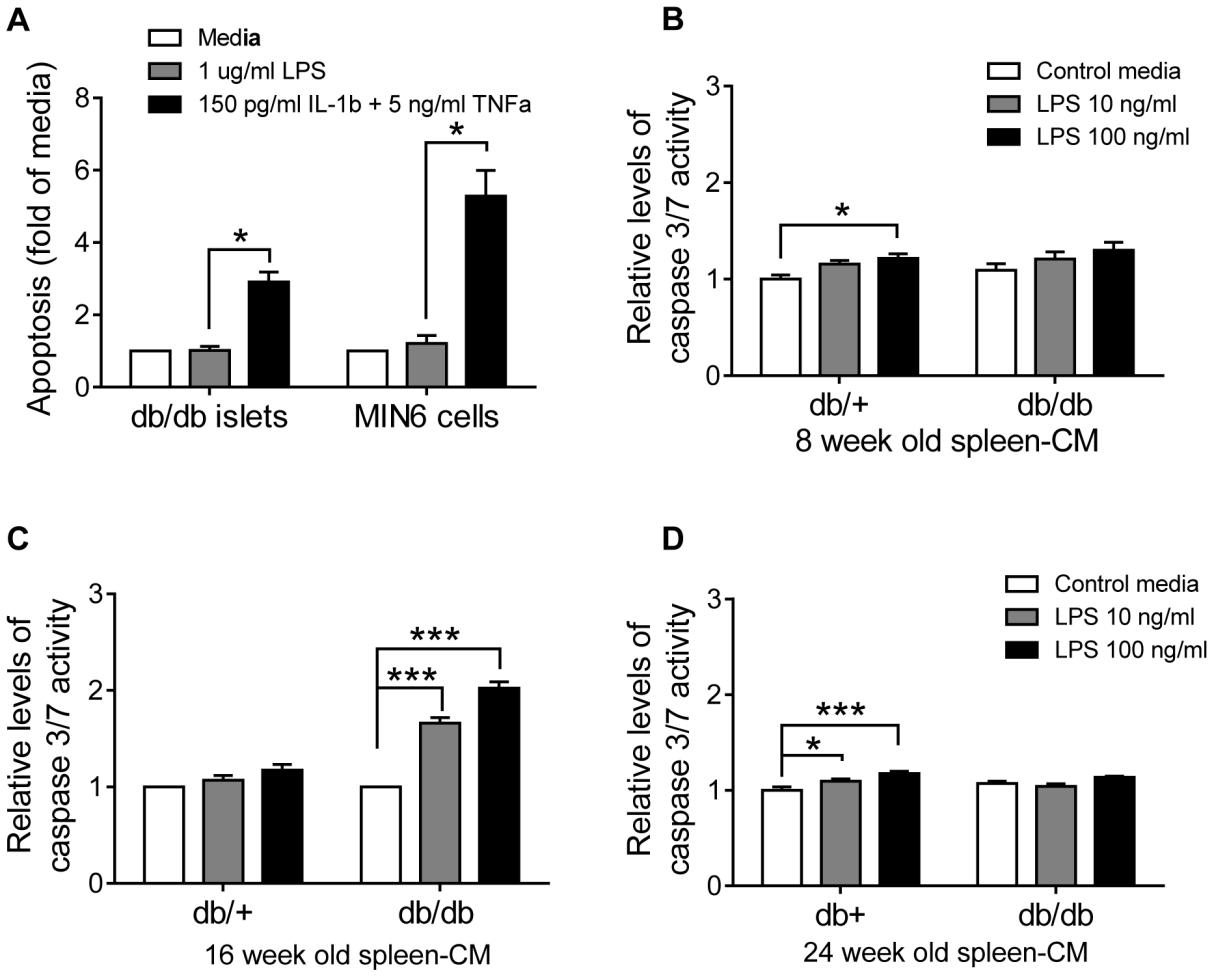
TLR4 induces β-cell apoptosis though release of soluble factors from macrophages. Db/db islets and MIN6 cells were stimulated with LPS or combined IL-1β and TNFα for 24 h and apoptosis was measured as cytosolic DNA-histone complexes (A). Media from 8-weeks (A), 16-weeks (B) or 24-weeks (C) old db+ or db/db TLR4 activated splenocytes were added to MIN6 cells for 24 h. MIN6 cell apoptosis was quantified as caspase3/7 activity. Data is shown as mean ±S.E.M. from three experiments. Statistical analysis by student T-test were *p<0.05; **p<0.01; ***p<0.001.

**Figure 8:**
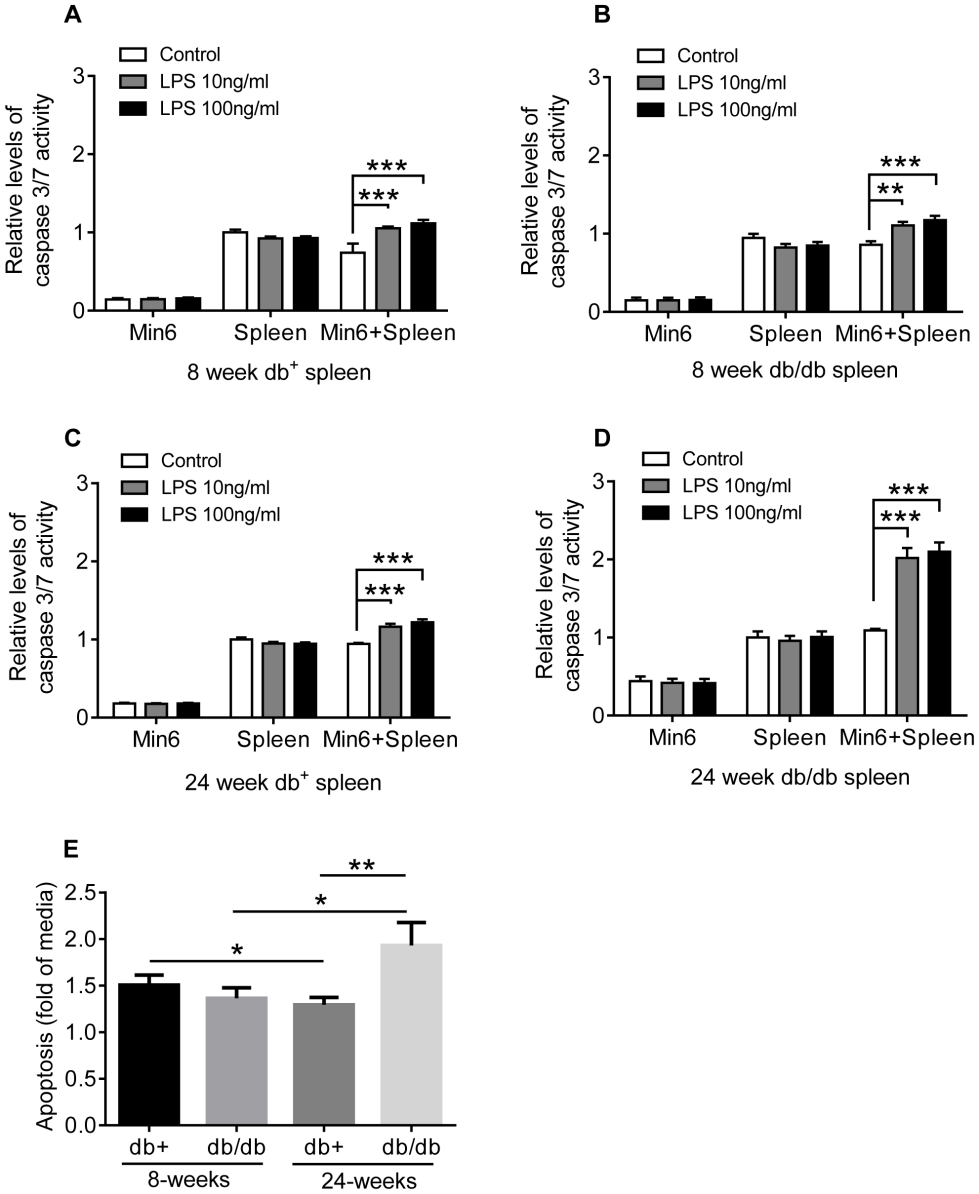
Diabetic splenocytes activated through TLR4 induce enhanced β-cell apoptosis. MIN6 cells were co-cultured with 8-week old db+ (A), 8-week old db/db (B), 24-week old db+ (C) or 24-week old db/db (D) for 24 h in media and then for an additional 24 h in the presence of absence of increasing concentration of LPS. Apoptosis was quantified as caspase3/7 activity. Data is shown as mean ±S.E.M. from three experiments. Statistical analysis by student T-test were *p<0.05; **p<0.01; ***p<0.001.

LPS had no direct effect on β-cell apoptosis, whereas a cytokine mixture of IL-1β and TNFα increased apoptosis 3-fold in the diabetic islets and 5-fold in the MIN6 cells after 24 h (Fig. 7A, 8A–D) and after 48 and 72 h (data not shown). Both IL-1β and TNFα are produced by macrophages in response to TLR4 (Fig. 6) and TLR4 positive macrophages are present in elevated numbers in the diabetic islet (Fig. 1). For these reasons, media from 8–24-week old TLR4 activated splenocytes were transferred to MIN6 cells to determine if the splenocyte derived cytokines were produced in concentrations required to induce apoptosis. Only a modest induction was observed when media from 8-week old and 28-week old diabetic or non-diabetic splenocytes were used (Fig. 7B, D). In sharp contrast, when media from 16-week old diabetic splenocytes were used, a strong apoptotic profile was obtained (Fig. 7C). This induction was not detected when age matched TLR4 activated healthy splenocytes were used (Fig. 7C).

These data demonstrate that TLR4 induces the production of soluble factors that are released by diabetic macrophages that lead to apoptosis of β-cells and islets.

### TLR4 Activated Diabetic Splenocytes Mediated Enhanced Apoptosis of β-cells

Direct cell contact is also known to induce pro-apoptotic mechanisms mediated by macrophages. To evaluate if cell-dependent apoptosis was also mediated in a TLR4-dependent manner, co-cultures between splenocytes and β-cells were conducted and induction of apoptosis measured.

Co-culture of splenocytes from both non-diabetic and diabetic 8-week old mice with MIN6 cells induced a moderate (<1.5-fold) pro-apoptotic response in the presence of TLR4 compared to co-cultures in media alone (Fig. 8A, B). A similar, but significantly lower profile with 1.3-fold TLR4 dependent apoptosis induction was evident in 24-week old compared to 8-week healthy splenocytes (Fig. 8C). Importantly, severely diabetic splenocytes from 24-week old db/db mice induced a 2-fold increase of apoptosis of MIN6 cells in co-culture experiments in presence of TLR4 (Fig. 8D). The level of TLR4 dependent apoptosis was significantly elevated compared to both 8-weeks diabetic splenocytes, but importantly also compared to age-matched healthy splenocytes (Fig. 8E).

**Figure 9:**
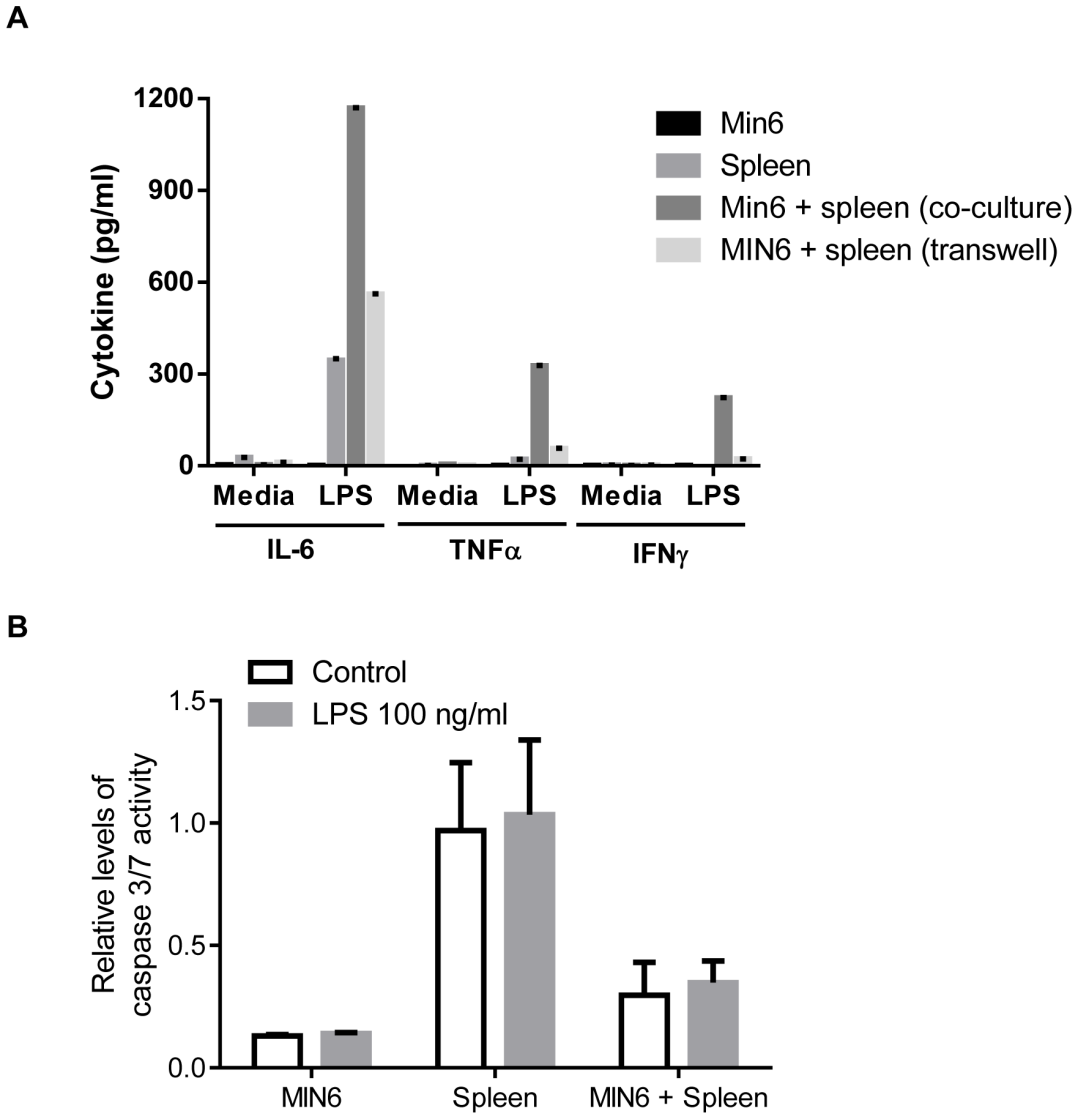
Cytokine induction in MIN6 cells, splenocytes, co-culture of MIN6 and splenocytes and transwell systems with MIN6 cells and splenocytes. (A). Induction of apoptosis in MIN6 cells cultured for 24 h and then for an additional 24 h in presence of LPS (B). Apoptosis was quantified as caspase 3/7 activity. Data is shown as mean +/− SEM from two experiments.

To evaluate if direct cell-contact is required to induce apoptosis in beta cells, MIN6 cells were added to the plate and the splenocytes were added in a transwell insert. Only when cultured in cell-contact dependent manner, significant levels of effector cytokines like TNFα and IFNγ were produced (Fig. 9A). More importantly, no induction of apoptosis was detected in the transwell systems (Fig. 9B).

Taken together, these data demonstrate that cell-contact dependent mechanisms contribute to the TLR4 dependent induction of apoptosis of β-cells probably by enhancing the capacity to produce effector cytokines in the macrophages. Interestingly, the data suggests that leukocytes from diabetic mice acquire enhanced capacity to mediate cell-contact dependent apoptosis of β-cells as a function of the time they have been in high glucose.

## Discussion

Toll-like receptor 4 (TLR4) has received much attention in the recent years due to its role in the development of insulin resistance in T2D. Apart from being involved in recognition of lipopolysaccharide, a constituent of gram negative bacteria cell wall, TLR4 can recognize endogenous ligands such as saturated free fatty acids thereby creating a direct link between the innate immunity and diet-induced obesity and insulin resistance [7], [8], [22]. In obese and Type 2 diabetic patients, the level of the TLR4 ligands, LPS and fatty acids are elevated [20] and recent studies demonstrate a resulting increase in TLR4 expression [12].

In accordance with other studies we could observe that both insulin producing cells and islet resident macrophages express TLR4 [7], [10], [21]. However, in contrast with a previous study we observed a tendency towards a general decrease of TLR4 expressing cells in the diabetic state [12]. However, the readout between the study by Ladefoged et al, and ours differs significantly in that we examine TLR4 protein levels whereas the previous study evaluated mRNA levels of TLR4 only. Thus, the discrepancies observed then suggest that TLR4 expression is regulated both on the mRNA level and on the protein level in diabetes and that the upregulation on the transcriptional level doesn’t correlate with the amount of protein finally expressed. The detailed analysis showed a clear trend towards decreased number of TLR4 positive insulin producing cells in diabetic mice that coincided with a statistically 3-fold increase of TLR4 positive macrophages locally in the islets. This relative reduction of TLR4 expressing insulin positive β-cells in the diabetic mice indicates that the TLR4 positive β-cells could be the cells most sensitive to undergo apoptosis in a local milieu characterized by a strong influx of TLR4 positive macrophages as in T2D [14] [13].

It has been suggested that the recruitment of these macrophages to the islets could be due to a FFA promoted increase of islet-derived chemokines [23]. Indeed, there is a significant increase of plasma FFA levels in mice on a high fat diet compared to mice on a regular chow diet and activation of TLR4 signaling by FFAs or LPS within pancreatic islets triggers the production of pro-inflammatory cytokines and chemokines that facilitate the local inflammatory process, leading to islet damage and rejection [5] [24]–[26]. However, whether these are produced by resident or infiltrating immune cells or by β cells themselves is debated. Several reports support an islet and β-cell origin of increased chemokine expression in T2D and associated animal models but do not rule out contribution from non-β-cells such as early infiltrated immune cells like macrophages [21], [24]. Our data shows that activation of the innate immune system through TLR4 indeed induces production of several chemokines *ex vivo*. Evaluation of freshly isolated diabetic islets supports that hyperglycemia by its own induces an intra-islet cytokine signature dominated by MCP-1 production.

The relevance of macrophages to the pathogenesis of T2D is just recently being recognized particularly through work demonstrating that the leukocytes indeed contribute to the disease progression and may indeed play an essential role [27]–[29]. In fact, prevention of macrophage activation precludes the development of obesity-related insulin resistance clearly placing the leukocyte arm as a key contributing factor in T2D [27]. TLR4 expression on macrophages, adipocytes and skeletal muscle cells is increased in obesity and essential for saturated fatty acid–induced activation of inflammation, meaning that potential activation of macrophage TLR4 by FFAs can be readily linked to insulin resistance [7], [30]. Most studies have addressed the role of TLR4 in tissues directly involved in metabolic actions of insulin, such as adipose tissue and muscles [8], [11], [28]–[31]. However, the role of TLR4 in the disease progression locally in the pancreatic tissue will almost certainly be important as well. Our findings support this hypothesis. We found that islets from diabetic mice are more potent in their pro-inflammatory response when exposed to the TLR4 agonist LPS and this correlates well with increased TLR4 expression on islet resident macrophages in these mice. Given that there is an elevated pro-inflammatory response upon LPS stimulation in db/db mice, despite the observed reduction of TLR4 positive insulin producing β-cells, suggests that the enlarged population of TLR4 positive resident macrophages found in diabetic mice are mainly responsible for driving the TLR4 dependent inflammatory processes occurring in diabetic pancreas. Another possibility is that LPS acts through an alternative receptor, TLR2 being a likely candidate. In fact, both TLR2 and TLR4 have been implicated in the signaling of LPS [32]. However, it has been established that TLR2-deficient macrophages display a normal production of pro-inflammatory cytokines in response to LPS, indicating that TLR2 alone is not involved in LPS response in mice. Conversely, TLR4-deficient mice lacked the response to LPS, demonstrating that TLR4 is required for LPS-induced pro-inflammatory responses [33]. Hence, it is unlikely that the cytokine response observed here is mediated through TLR2.

A hallmark of diabetes is reduced β-cell function measured as reduction of glucose stimulated insulin secretion (GSIS) [34]. Several cytokines have been demonstrated to participate in this reduced GSIS [35]. Our data that TLR4 activation of islets induces enhanced production of several cytokines suggests that this mechanism indeed may be in function also *in vivo* as several of the cytokines are produced in significant levels. Furthermore, direct evidence that TLR4 dependent mechanisms indeed contribute to decline in β-cell function was demonstrated when addition of LPS to islets showed a clear reduction of GSIS. Moreover, TLR4 expression on pancreatic β-cells has been shown to correlate with loss of cell viability and to increase upon LPS stimulation [10]. Hence, the observed decline of TLR4 positive insulin producing β-cells already in 8 week old db/db mice could reflect a TLR4 mediated apoptotic cell death initiated by the significant elevated FFA levels occurring at this age [36]. Surprisingly, we found that healthy islets expressed TLR4 even at higher levels than in the diabetic littermates. Importantly, in the healthy pancreas TLR4 expression was almost exclusively restricted to β-cells as almost no infiltrating macrophages were present within the healthy islets. As LPS stimulation of healthy islets failed to induce significant cytokine production despite the relatively high TLR4 expression, the local innate immunity activated by TLR4 clearly appears to require presence of invading macrophages. This strengthens our conclusion that TLR4 expression on insulin producing cells alone is not sufficient for pancreatic islet cell dysfunction and death. Rather, it clearly places the recruited macrophages as a key cell type essential for the islet pathology in type 2 diabetes.

Macrophages are highly plastic cells that arise from circulating monocytes that have entered target tissues and gained the phenotypic and functional attributes of their tissue of residence. Cucak et al recently demonstrated that CD68^+^F4/80^−^ macrophages are significantly invading diabetic islets in db/db mice. Moreover, macrophages express elevated levels of pro-inflammatory cytokines and express a phenotypic signature (high surface levels of CD11c) associated with the classically activated M1-like macrophages [18]. Recently, TLR4 signaling was shown to play a direct role in mediating adipose tissue macrophage phenotype in diet-induced obesity and may shed some light on why TLR4 deficiency results in decreased adipose tissue inflammation despite that the prevalence of ATMs is not reliably reduced [31] [7]. Hence the M1/M2 paradigm is thought to play a major role in β cell dysfunction and obesity. Shi et al show that mice on a high fat diet display an induced expression of macrophage marker F4/80 in adipose tissue compared to mice on a regular chow diet, and that this was greatly reduced in TLR4 knockout mice [7]. Here, we extend this into a diabetic situation as we demonstrated significantly increased TLR4 positive CD68^+^F4/80^+^ and CD68^+^F4/80^−^ islet resident macrophages in diabetic db/db mice compared to non-diabetic db/+ mice. Importantly, this did not correspond to the systemic population in spleen of these mice. This discrepancy between the systemic macrophage compartment and the local compartment clearly suggests that the macrophages undergo final maturation in the local environment where they acquire their effector functions.

In spleen, the number of TLR4 positive CD68^+^F4/80^+^ macrophages was comparable between diabetic and healthy mice and decreased with age. In contrast, the number of TLR4 positive CD68^+^F4/80^−^ macrophages increased with age and there was a trend implying that this population may be expanded in diabetic mice. Recently, we demonstrated that the db/db mice have elevated systemic plasma levels of several cytokines such as MCP-1, KC, M-CSF, GM-CSF and TNFα [18]. Importantly, the TLR4 positive CD68^+^F4/80^−^ macrophages were primarily responsible for the production of the pro-inflammatory cytokines IL-6 and IL-1β, upon TLR4 stimulation. Both these cytokines have been proposed as key mediators of insulin resistance [24]. It has been postulated that local secretion of low amounts of IL-1β by islet cells, has a vital role in the upholding of β-cell function, while lasting elevated pathological levels of IL-1β, feasibly produced by infiltrating immune cells, result in decreased β-cell function and mass in diabetes [24]. This is further supported by that the expression of IL-1β is over one hundred-fold higher in β cells in patients with T2D. Similarly, patients with T2D show increased systemic levels of IL-6 [37] and islets isolated from type 2 diabetic animal models produce increased amounts of IL-6 [13]. Additionally, sustained treatment of human and rodent islets with IL-6 impairs glucose stimulated insulin secretion [24].

In line with this, while the capacity to produce cytokines was similar in early age between the diabetic and non-diabetic splenocytes, the diabetic splenocytes acquired enhanced capacity to produce cytokines at 16-weeks of age. Most interestingly, the enhanced cytokine production was transient and normalized to the level observed in non-diabetic animals at 24-weeks of age, an age that coincides with the compensatory peak production of insulin in the β-cells [14]. In fact, it is established that in established disease in diabetic mice a general impairment of the immune system associated with reduced phagocytic capacity and a defective oxidative burst, similar to that found in human diabetic patients is evident [38]. This defect in the immune system is translated in diabetic patients to an enhanced susceptibility to infections, especially from gram negative bacteria [39], [40]. A similar defect in bacterial clearance has been demonstrated in old leptin receptor deficient db/db mice that indeed show weakened bacterial clearance of gram negative bacterial strains compared to heterozygote littermates [39], [40]. Moreover, a decline in LPS responsiveness in peritoneal macrophages from db/db mice with characteristics relevant to developed T2D has previously been reported [41]. Hence, the immune alterations associated with aging in db/db mice seem to have a profile resembling that of the cellular immune defects associated with T2D, i.e a less reactive systemic immunity particularly in the Toll-pathways.

T2D diabetes is characterized by a defect function of the β-cells followed by enhanced level of apoptosis which leads to the establishment of the disease [42]. Cytokine mediated cell death of the β-cells has been suggested and shown both in human and murine islets as well as in isolated β-cell lines [43]–[46]. As we have demonstrated that there is enhanced TLR4 dependent cytokine production in the diabetic islets and elevated accumulation of macrophages in the islet structure it is tempting to speculate that the cytokines released from the TLR4 activated macrophages contribute to the enhanced apoptosis. Indeed, when the media from splenocytes activated with TLR4 was added to β-cells a clear apoptotic profile was evident, but only in media from 16-week old diabetic splenocytes. This clearly demonstrates that factors released by the TLR4 activated macrophages are produced in sufficient amounts to induce apoptosis of the β-cells. This observation is in line with previous demonstration that macrophages can induce bystander apoptosis by release of soluble factors e.g. TNFα and FasL [47], [48]. It is not clear from our study which factor is responsible for the bystander apoptosis, but the significantly enhanced level of the known pro-apoptotic TNFα cytokine suggests this to be a likely candidate. Interestingly, when splenocytes were co-cultured with β-cells a clear pro-apoptotic signature was obtained also with diabetic splenocytes from 24-weeks old mice. This suggests that the diabetic, but not healthy macrophages have acquired additional mechanisms allowing them to induce β-cell apoptosis that is cell-contact dependent. This type of cell-contact dependent apoptosis mediated by macrophages has been described in several other pathological conditions such as atherosclerosis that often coincide in T2D [49]. With the enhanced number of macrophages expressing TLR4 showing an activated phenotype and the previously demonstrated co-localization of macrophages to β-cells in diabetes, the present study further emphasis the emerging role of auto-inflammation in the progression of T2D [13].

**Figure 10:**
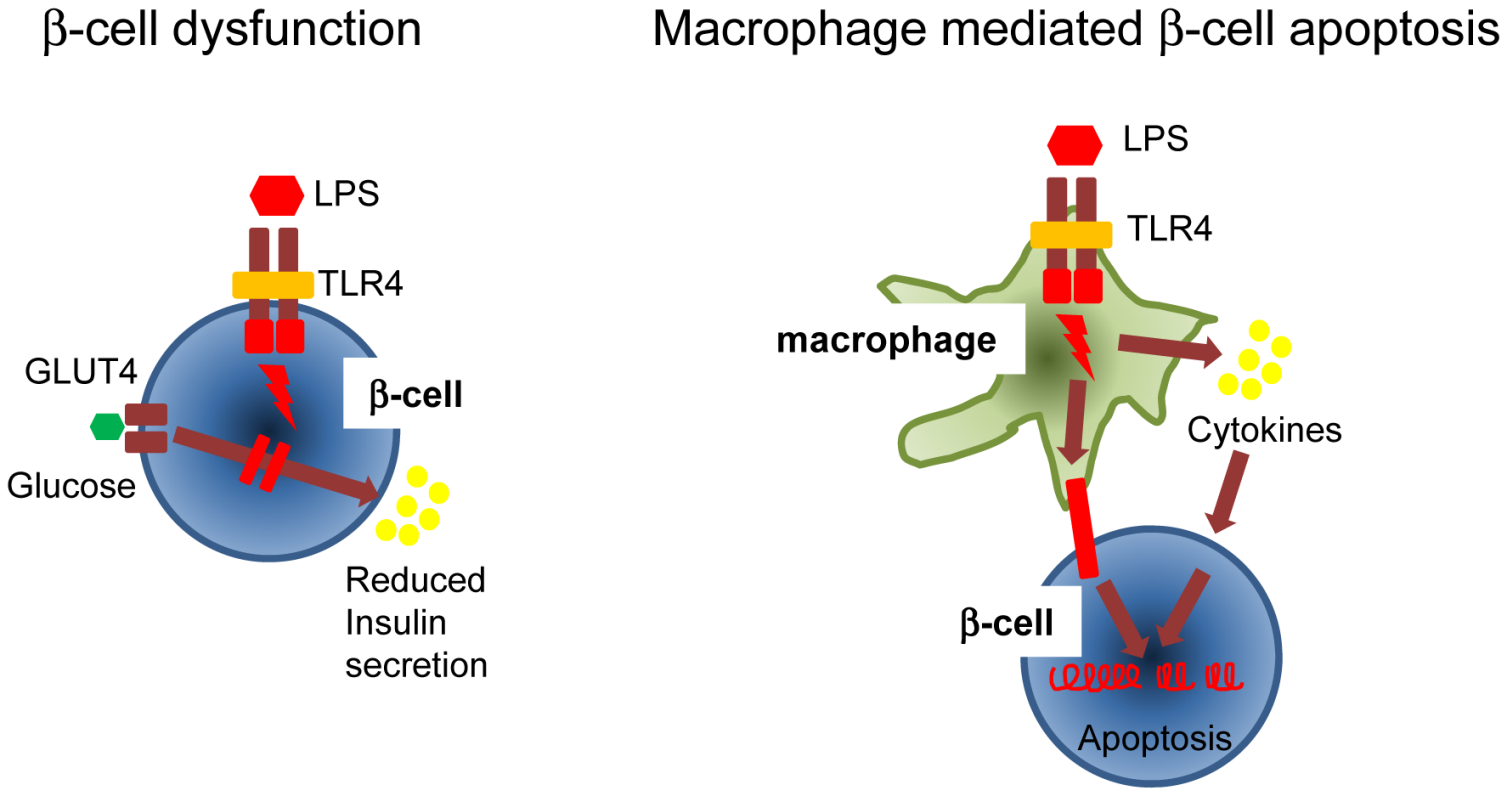
Hypothesis of TLR4 mechanisms in diabetic islet dys-function.

Taken together, herein we show that in the diabetic islet the β-cells express TLR4 and an enhanced TLR4 positive macrophage population is present. Further, the diabetic islets showed an enhanced TLR4 dependent cytokine production upon challenge. Most importantly, β-cell dysfunction was demonstrated with reduced glucose stimulated insulin secretion and both cell-contact dependent and independent apoptosis of β-cells were shown to be TLR4 dependent (Fig. 10). Thus, the data generated herein supports the hypothesis that inflammatory pathways mediated through the innate immunity contribute to development of T2D.

## Conclusions

This study has demonstrated that in type 2 diabetes a significant immigration of TLR4 positive macrophages occurs into the pancreatic islet. This leads to augmented production of cytokines within the diabetic islets in a TLR4 dependent manner. Both TLR4 derived soluble factors from activated macrophages as well as direct cell-contact mechanisms provided by macrophage-β-cell interactions result in significant apoptosis of β-cells. TLR4 activation directly results in β-cell dysfunction measured by diminished glucose induced insulin secretion. Thus, this study provides novel mechanistical understanding of the role the innate immunity plays in type 2 diabetes.

## Supporting Information

Figure S1 Chemokine secretion in mouse islets. 10 NMRI islets were stimulated with as indicated for 24 h. The supernatants were analyzed with milliplex assay for secretion of selected chemokines. Data shown is mean values +/− SEM from three independent experiments. p*<0.05, p**<0.01, p***<0.001, (one-way ANOVA).
(TIF)
