# Supplementary figures and images for: Macrophage Contact Dependent and Independent TLR4 Mechanisms Induce β-Cell Dysfunction and Apoptosis in a Mouse Model of Type 2 Diabetes
Source: PLoS One. 2014 Mar 3;9(3):e90685. doi: 10.1371/journal.pone.0090685 (PMC3940939; doi:10.1371/journal.pone.0090685)

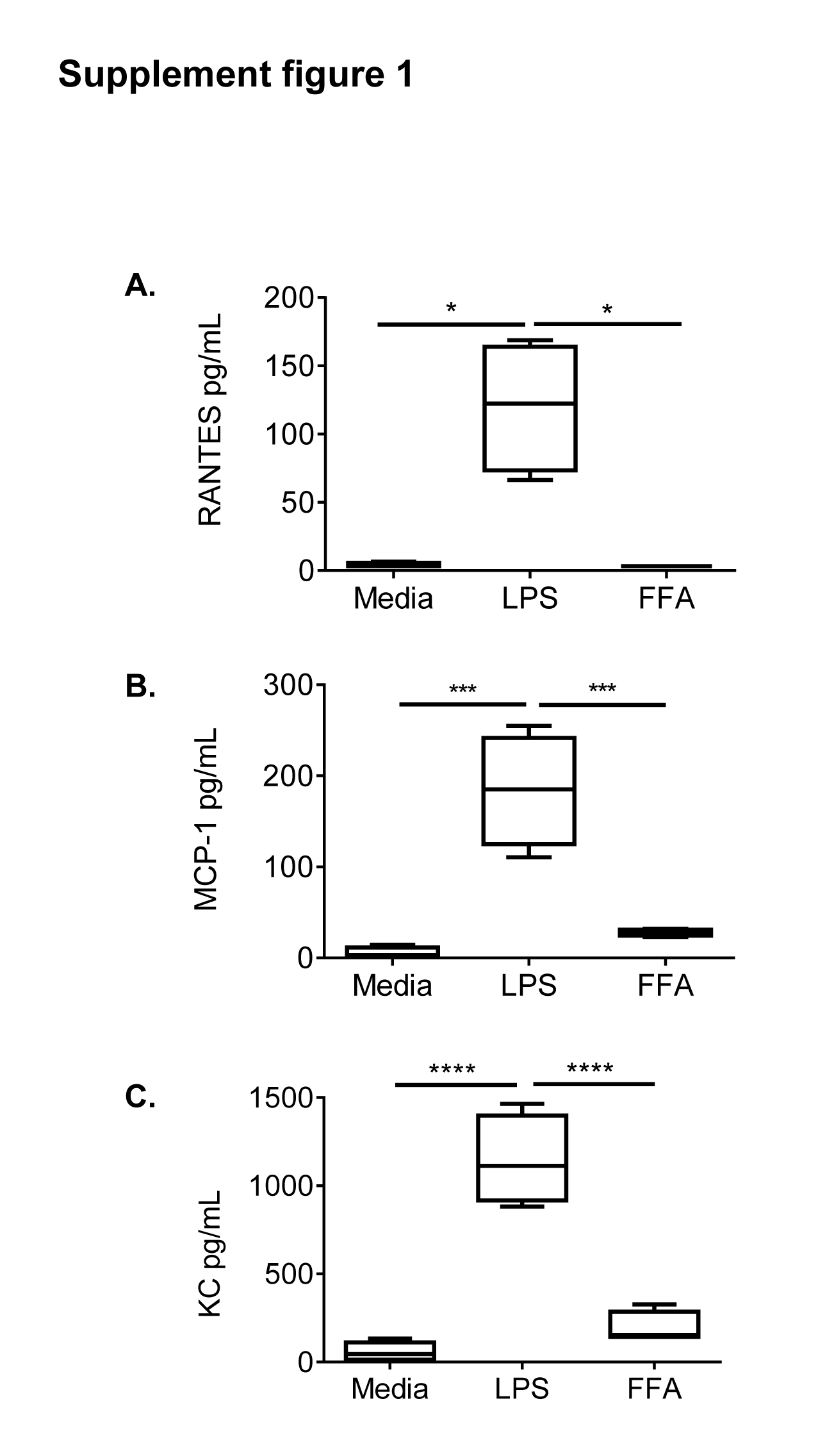

Supplement: Figure S1 — Chemokine secretion in mouse islets. 10 NMRI islets were stimulated with as indicated for 24 h. The supernatants were analyzed with milliplex assay for secretion of selected chemokines. Data shown is mean values +/− SEM from three independent experiments. p*<0.05, p**<0.01, p***<0.001, (one-way ANOVA). (TIF) [file pone.0090685.s001.tif]
